# Bacterial and fungal community composition and community-level physiological profiles in forest soils

**DOI:** 10.1371/journal.pone.0284817

**Published:** 2023-04-20

**Authors:** Takashi Kunito, Shota Hibino, Hirotaka Sumi, Kozue Sawada, Ho-Dong Park, Kazunari Nagaoka

**Affiliations:** 1 Department of Environmental Science, Faculty of Science, Shinshu University, Matsumoto, Japan; 2 Department of Biological Chemistry, College of Bioscience and Biotechnology, Chubu University, Kasugai, Aichi, Japan; 3 Graduate School of Bioagricultural Sciences, Nagoya University, Nagoya, Aichi, Japan; 4 Central Region Agricultural Research Center, NARO, Tsukuba, Ibaraki, Japan; ICAR-Indian Institute of Soil Science, INDIA

## Abstract

We characterized the potential functioning and composition of the bacterial and fungal communities in the O and A horizons of forest soils using community-level physiological profile (CLPP) based on BIOLOG analysis, and polymerase chain reaction–denaturing gradient gel electrophoresis (PCR-DGGE) analysis of 16S and 18S rDNA fragments, respectively. In addition, relationships between the potential functioning and the community composition in each horizon, and between the O and A horizons, were assessed using Procrustes analysis. For the bacterial and fungal communities, the CLPP and DGGE profile were clearly separated between the O and A horizons in a principal coordinate analysis except for the fungal CLPP. No significant links for CLPP and DGGE profile between the O and A horizons were observed for either bacterial or fungal communities, suggesting that different factors had considerable influence on the microbial communities between the O and A horizons. Significant couplings between bacterial and fungal DGGE profiles (*p* <0.05 for O horizon; *p* <0.01 for A horizon), and between bacterial and fungal CLPPs (*p* = 0.001 for O horizon; *p* <0.01 for A horizon), were observed in the O and A horizons, implying that common factors strongly influenced the bacterial and fungal communities in each horizon. Although a significant correlation was observed between bacterial community composition and the potential functioning in the A horizon (*p* <0.01), such a correlation was not observed for the fungal community in the A horizon, and for the bacterial and fungal communities in the O horizon. This finding suggested that potential functioning, which would reflect only rapidly growing microorganisms, was not strongly associated with the composition of the entire microbial community. Further studies are needed to unravel the factors shaping the composition and functioning of microbial communities in forest soils.

## Introduction

80%–90% of the processes in soils may be mediated by microorganisms [1]. Therefore, the composition and functioning of the soil microbial communities have significant implications for carbon (C) and nutrient cycling, especially in forest soils rich in organic matter. The composition of plant litter (e.g., the relative proportions of cellulose and lignin) may affect the structure and functioning of microbial communities [2]. In addition, soil properties, such as nutrient concentrations and pH, influence these aspects of the microbial communities [3–5]. However, it remains important to develop an improved understanding of (i) the factors that strongly influence the composition and functioning of microbial communities, (ii) the relationships in potential functioning and community composition between the soil O and A horizons, and (iii) the degree to which microbial community diversity is translated into functional diversity in forest soils.

BIOLOG microplate technique and polymerase chain reaction–denaturing gradient gel electrophoresis (PCR-DGGE) analysis of rDNA fragments have often been used to characterize potential functioning and community composition of microbial community, respectively [6]. In response to criticism that community-level physiological profile (CLPP) based on the BIOLOG microplate provide a biased representation of the functional ability of culturable bacteria capable of rapidly growing on substrates in the BIOLOG plate [7–9], Lladó and Baldrian suggested that CLPP was able to evaluate the functional potential of the fast-growing copiotrophic bacteria [9], which are active or potentially active bacteria that largely contribute to nutrient cycling in soils [10–12]. For fungal CLPPs, the limitation that the BIOLOG plate selects only culturable microorganisms is minor because the majority of fungi are culturable except for obligate symbionts [13]. To date, many studies have supported the utility of CLPP for studying the potential functioning of the microbial community and comparing microbial communities in different samples [14–20]. Although DGGE analysis does not provide direct phylogenetic information and underestimates total microbial diversity, it is a rapid and inexpensive method that quantitatively detects differences in the diversity and composition of microbial communities, which is comparable to those of high-throughput sequencing [21]. Using the BIOLOG microplate technique and PCR-DGGE analysis of 16S and 18S rDNA fragments, we characterized two attributes of the bacterial and fungal communities in the O and A horizons of forest soils, namely their potential functioning and composition.

In this study, the following three hypotheses were tested. We hypothesized that microbial community composition and potential functioning will differ distinctly between the O and A horizons because of differences in organic matter composition and pH (Hypothesis 1), but that these microbial characteristics would covary between the O and A horizons owing to the possible linkage in organic matter characteristics and pH between the two horizons at each site. Thus, differences in the O horizon between sites are accompanied by corresponding differences in the A horizon between sites for both community composition and potential functioning (Hypothesis 2). In addition, we hypothesized that potential functioning of the microbial community is associated with the community composition (Hypothesis 3).

## Materials and methods

### Soils

Litter and soil samples were collected from the O and A horizons, respectively, at each of 12 forest sites (35.81°N–36.34°N, 137.81°E–137.99°E) in Nagano Prefecture, Japan, in November, 2009 (*n* = 6 for Andosols; *n* = 6 for Cambisols). The altitude of the sampling sites ranged from 700 to 2,045 m. Most of the sites were situated in conifer forests (vegetation at most sites was *Larix kaempferi*, with *Pinus densiflora* and *Cryptomeria japonica* at some sites). Because the vegetation was the same at most sites, we did not examine the effect of vegetation on microbial community composition and potential functioning. At each site, samples were collected from five plots, and then were pooled and mixed to form a composite sample and sieved through a 2 mm mesh. The litter and soil samples were stored at −20 °C for DNA extraction, and at 4 °C for BIOLOG and microbial biomass C measurements. A portion of the soil sample was air-dried for chemical analyses, whereas a portion of the litter sample was dried at 70 °C and then ground to powder using a blender (Osaka Chemical WB-1, Osaka, Japan). For pH measurement, the dried litter samples were not ground.

The pH was measured from a soil–water suspension (1:2.5, w/v) or a litter–water suspension (1:50, w/v) with a glass electrode [22]. Organic C and total nitrogen (N) contents were determined by dry combustion using an elemental analyzer (Thermo Finnigan Flash EA1112, Waltham, MA, USA) [22]. The ground litter sample was fractionated into water-soluble polysaccharide, hemicellulose, cellulose, lignin, and lipids at Createrra, Inc. (Tokyo, Japan) using the proximate analytical method of Waksman and Stevens [23] with some modifications [22]. Organically bound (Al_p_ and Fe_p_) and non-crystalline and organically bound forms (Al_o_ and Fe_o_) of aluminum (Al) and iron (Fe) were extracted with 0.1 M sodium pyrophosphate (pH 10) and 0.2 M acid ammonium oxalate (pH 3), respectively [24]. Aluminum and Fe were analyzed using flameless and flame atomic absorption spectrometry (Perkin Elmer 5100 PC, Tokyo, Japan), respectively. All data are expressed on a dry weight basis. All the analyses, including the microbial ones described below, were performed in 2009 and 2010.

### PCR-DGGE analysis of 16S and 18S rDNA fragments

We analyzed the composition of the bacterial and fungal communities in the litter and soil samples by PCR-DGGE [25, 26]. Total soil DNA was extracted from 0.4 g of each sample using the FastDNA SPIN Kit for Soil (MP Biomedicals, Illkirch-Graffenstaden, France) in accordance with the manufacturer’s instructions. Given the difficulty of extracting DNA from the Andosol samples, heat-treated skim milk (treated at 115 °C for 5 min) was added before cell lysis [27] to inhibit DNA adsorption to humic acid and allophane in the soil [28]. However, DNA could not be extracted from two samples from the A horizon of Andosols. Thus, four samples from the A horizon of Andosols were used for the following analysis.

The bacterial 16S rDNA fragment for DGGE analysis was amplified by PCR with the primer set 968f-GC and 1378r. After initial denaturation at 94 °C for 2 min, 34 amplification cycles were performed (denaturation at 94 °C for 15 s, annealing at 55 °C for 30 s, and extension at 68 °C for 30 s). The fungal 18S rDNA fragment was amplified with the primer set NS1 and GCFung. After denaturation at 94 °C for 2 min, 30 amplification cycles were performed (denaturation at 94 °C for 15 s, annealing at 50 °C for 30 s, and extension at 68 °C for 30 s). The PCR products were purified using the QIAquick PCR Purification Kit (Qiagen, Valencia, CA, USA).

The DGGE analysis was performed with a DCode Universal Mutation Detection System (BioRad Laboratories, Hercules, CA, USA). For bacterial community analysis, a 6% polyacrylamide gel with a linear denaturing gradient range of 50%–70% was used to separate the 16S rDNA PCR products (100% denaturant is defined as 7 M urea and 40% [v/v] formamide). Each lane was loaded with 200 ng purified PCR products. The PCR products were separated by electrophoresis at 58 °C and 50 V for 18 h. For fungal community analysis, a 7% polyacrylamide gel with a linear denaturing gradient ranging from 20% to 45% was used, and the running condition was 60°C and 50 V for 20 h. In the bacterial and fungal DGGE analyses, DGGE Markers III and IV (Nippon Gene, Toyama, Japan) were used as the molecular markers, respectively [29]. After electrophoresis, the gels were stained with SYBR Green I Nucleic Acid Gel Stain (Cambrex Bio Science, Rockland, ME, USA) and scanned with a ChemiDoc XRS system (BioRad Laboratories). Gel images were analyzed using Fingerprinting II software (BioRad Laboratories).

Community diversity was evaluated using the number of DGGE bands (species richness) and the Shannon–Wiener diversity index (*H′*). *H′* was calculated using the following equation: *H′* = −Σ *p*_i_ (ln *p*_i_), where *p*_i_ is the proportion of the intensity of each band to the sum of intensity per profile. The proportion of the intensity of each band was also used in the principal coordinate analysis (PCoA).

### BIOLOG and microbial biomass C measurements

CLPPs of bacteria and fungi, based on the BIOLOG ECO plate and SFN2 plate (BIOLOG, Hayward, USA), respectively [30, 31], were determined to assess the potential functioning and functional diversity [6, 7]. The CLPP was conducted within one week after sampling. For the fungal CLPP, the inoculation solution was supplemented with antibiotics (10 mg L^−1^ streptomycin sulfate and 5 mg L^−1^ chlortetracycline) to inhibit bacterial growth. The ECO plates were incubated at 28 °C for 72 h, and absorbances at 595 nm and 750 nm were measured with a microplate reader (Model 680XR, BioRad). After correcting for the absorbances at 595 nm and 750 nm in each well at 0 h and the water well at 72 h, the value for each well used for subsequent analysis was the 595 nm absorbance (color development plus turbidity) minus the 750 nm absorbance (turbidity) at 72 h [32]. The SFN2 plates were incubated for 168 h; the absorbance was measured at 750 nm and corrected for the readings in each well at 0 h and in the water well at 168 h. Well optical density values of less than 0.1 were set to zero. The overall color development in each plate was expressed as the average well color development (AWCD). Potential functional diversity was calculated using the Shannon–Wiener index (*H*′): *H*′ = −Σ *p*_*i*_ (ln *p*_*i*_), where *p*_*i*_ is the proportion of the absorbance value of the *i*th substrate to the sum of absorbance values of all substrates in a plate. The proportion of the absorbance value of each substrate was also used in the PCoA.

Microbial biomass C in the soils was measured using the chloroform fumigation–extraction method as described previously [33, 34]. Soil was fumigated with ethanol-free chloroform for 24 h at 25°C and then extracted with 0.5 M K_2_SO_4_ for 30 min. The organic C content in the extracts was measured with an organic C analyzer (Shimadzu TOC-V, Kyoto, Japan). Soil microbial biomass C (C_mic_) was calculated using a conversion factor (*k*_EC_ = 0.49) as follows [35]: C_mic_ (μg g^−1^) = *E*_C_ / *k*_EC_, where *E*_C_ = (amount of C (μg g^−1^) extracted by 0.5 M K_2_SO_4_ from fumigated soil)–(amount of C (μg g^−1^) extracted by 0.5 M K_2_SO_4_ from non-fumigated soil).

### Statistical analyses

Welch’s *t*-test was used to detect a significant difference between the means of two samples. Scheffé’s test together with one-way analysis of variance (ANOVA) were used to evaluate significant differences for multiple-group comparisons. Pearson correlation analysis was performed to measure the strength of the associations between variables. These analyses were conducted using BellCurve for Excel (Social Survey Research Information, Tokyo, Japan). The PCoA was performed on the data from the DGGE analysis and CLPP using Bray–Curtis dissimilarity matrices. Procrustes analysis, a method of comparing two sets of configurations, was performed to assess the extent that the CLPP and DGGE data yielded similar results with respect to PCoA ordinations among samples with 999 permutations. The PCoA and Procrustes analyses were conducted with the *vegan* package in R (version 4.1.2).

## Results

### DGGE analysis of bacterial and fungal communities

Bacterial richness (i.e., the number of bands in a 16S rDNA DGGE fingerprint) in the A horizon was significantly higher in the Andosols than in the Cambisols, but no significant difference was observed in the O horizon between the Andosols and Cambisols (Table 1). Both soil types exhibited significantly higher bacterial richness in the A horizon than in the O horizon (*p* <0.05). Bacterial *H*′ was also significantly greater in the A horizon than in the O horizon (*p* <0.05), whereas no significant difference was observed between the soil types in each of the O and A horizons. Only a significant negative influence of hemicellulose content on bacterial *H*′ in the O horizon (*p* <0.05; Table 2) and a significant positive influence of Fe_p_ content on bacterial *H*′ in the A horizon (*p* <0.05; Table 3) were observed.

**Table 1:** Properties of samples used in this study.

	Andosols (*n* = 6)		Cambisols (*n* = 6)	
	O horizon	A horizon	O horizon	A horizon
pH	5.14 ± 0.32	4.68 ± 0.35	5.39 ± 0.49	5.29 ± 1.09
Organic C (mg g^−1^)	505 ± 35 a	101 ± 32 b	514 ± 32 a	48 ± 30 b
Total N (mg g^−1^)	10.1 ± 4.1 a	6.0 ± 1.7 ab	8.1 ± 5.8 ab	2.9 ± 1.2 b
Water-soluble polysaccharide (mg g^−1^)	67 ± 17 b		134 ± 36 a	
Hemicellulose (mg g^−1^)	189 ± 40		221 ± 92	
Cellulose (mg g^−1^)	165 ± 46		156 ± 36	
Lignin (mg g^−1^)	365 ± 49 a		272 ± 50 b	
Lipids (mg g^−1^)	74 ± 54		111 ± 73	
Al_p_ (mg g^−1^)		46.3 ± 29.3 a		3.8 ± 2.6 b
Al_o_ (mg g^−1^)		52.8 ± 23.0 a		7.6 ± 2.9 b
Fe_p_ (mg g^−1^)		13.9 ± 9.8		5.2 ± 5.4
Fe_o_ (mg g^−1^)		21.1 ± 11.6 a		2.5 ± 1.4 b
Biomass C (µg g^−1^)		955 ± 314		615 ± 555
Band number of 16S rDNA DGGE	14.7 ± 2.9 bc	27.3 ± 3.4 a	12.8 ± 2.1 c	19.3 ± 3.9 b
*H’* of 16S rDNA DGGE	2.06 ± 0.32 b	2.86 ± 0.08 a	2.01 ± 0.28 b	2.53 ± 0.16 a
Band number of 18S rDNA DGGE	10.0 ± 2.8	8.5 ± 2.1	7.5 ± 1.5	9.8 ± 2.7
*H’* of 18S rDNA DGGE	1.94 ± 0.32	1.71 ± 0.33	1.53 ± 0.23	1.90 ± 0.29
AWCD of ECO plate for bacteria	0.51 ± 0.20	0.39 ± 0.12	0.26 ± 0.15	0.41 ± 0.15
*H’* of ECO plate for bacteria	3.18 ± 0.12	3.06 ± 0.13	2.97 ± 0.26	3.05 ± 0.16
AWCD of SFN2 plate for fungi	0.44 ± 0.20	0.37 ± 0.05	0.30 ± 0.15	0.33 ± 0.16
*H’* of SFN2 plate for fungi	4.25 ± 0.13	4.17 ± 0.10	3.99 ± 0.33	3.88 ± 0.52

*n* = 4 for DGGE data in A horizon of Andosols because DNA could not be extracted.

Row numbers without a letter or followed by the same letter are not significantly different (*p* >0.05).

**Table 2:** Pearson correlation coefficients between microbial and litter properties in O horizon (*n* = 12).

	Band number of 16S rDNA DGGE	*H’* of 16S rDNA DGGE	Band number of 18S rDNA DGGE	*H’* of 18S rDNA DGGE	AWCD of ECO plate	*H’* of ECO plate	AWCD of SFN2 plate	Altitude	pH	Organic C	Total N	C/N	Water-soluble polysaccharide	Hemicellulose	Cellulose	Lignin	Lipids
Band number of 16S rDNA DGGE	—	—	—	—	—	—	—	-0.051	-0.036	0.072	-0.09	0.168	-0.309	-0.494	0.284	0.301	0.113
*H’* of 16S rDNA DGGE	0.859***	—	—	—	—	—	—	-0.370	0.257	-0.019	-0.13	0.338	-0.052	-0.578*	0.300	0.084	0.276
Band number of 18S rDNA DGGE	0.032	-0.018	—	—	—	—	—	0.597*	-0.315	-0.284	0.338	-0.326	-0.538	-0.029	0.146	0.548	-0.373
*H’* of 18S rDNA DGGE	0.093	-0.036	0.968***	—	—	—	—	0.705*	-0.357	-0.260	0.342	-0.370	-0.620*	0.020	-0.005	0.662*	-0.416
AWCD of ECO plate for bacteria	0.296	0.285	0.694*	0.688*	—	—	—	0.359	-0.045	-0.589*	0.531	-0.473	-0.701*	-0.098	0.348	0.714*	-0.522
*H’* of ECO plate for bacteria	0.205	0.239	0.382	0.331	0.860***	—	—	0.040	0.009	-0.493	0.497	-0.430	-0.592*	-0.065	0.445	0.537	-0.462
AWCD of SFN2 plate for fungi	0.567	0.407	-0.029	-0.001	0.471	0.554	—	-0.124	0.050	-0.340	0.177	-0.201	-0.235	-0.147	0.500	0.179	-0.216
*H’* of SFN2 plate for fungi	0.439	0.485	0.259	0.203	0.749**	0.915***	0.719**	-0.183	0.073	-0.367	0.361	-0.224	-0.448	-0.281	0.527	0.403	-0.246

*, *p* <0.05;

**, *p* <0.01;

***, *p* <0.001.

**Table 3:** Pearson correlation coefficients between microbial and soil properties in A horizon (*n* = 12 except for *n* = 10 of DGGE data).

	Band number of 16S rDNA DGGE	*H’* of 16S rDNA DGGE	Band number of 18S rDNA DGGE	*H’* of 18S rDNA DGGE	AWCD of ECO plate	*H’* of ECO plate	AWCD of SFN2 plate	*H’* of SFN2 plate	Altitude	pH	Organic C	Total N	C/N	Al_p_	Al_o_	Fe_p_	Fe_o_
Band number of 16S rDNA DGGE	—	—	—	—	—	—	—	—	0.249	-0.288	0.299	0.472	-0.373	0.484	0.576	0.496	0.452
*H’* of 16S rDNA DGGE	0.971***	—	—	—	—	—	—	—	0.345	-0.342	0.419	0.564	-0.259	0.561	0.596	0.656*	0.592
Band number of 18S rDNA DGGE	-0.415	-0.389	—	—	—	—	—	—	-0.266	0.197	-0.173	-0.222	0.254	-0.203	-0.187	-0.150	-0.165
*H’* of 18S rDNA DGGE	-0.412	-0.389	0.962***	—	—	—	—	—	-0.348	0.153	-0.198	-0.236	0.217	-0.220	-0.202	-0.162	-0.205
AWCD of ECO plate for bacteria	-0.046	-0.036	-0.164	-0.248	—	—	—	—	0.163	-0.429	0.029	-0.069	0.361	0.142	0.169	0.087	0.083
*H’* of ECO plate for bacteria	0.285	0.298	-0.136	-0.143	0.853***	—	—	—	-0.017	-0.377	-0.054	-0.078	0.221	0.112	0.142	0.330	0.267
AWCD of SFN2 plate for fungi	0.380	0.279	-0.746*	-0.613	0.277	0.418	—	—	-0.077	-0.175	-0.103	-0.058	-0.179	0.203	0.204	0.119	0.064
*H’* of SFN2 plate for fungi	0.503	0.469	-0.260	-0.180	0.597*	0.744**	0.613*	—	0.221	-0.698*	0.258	0.258	0.246	0.272	0.330	0.405	0.390
Biomass C	-0.047	0.014	-0.144	-0.198	0.271	0.025	-0.072	0.244	0.932***	-0.725**	0.861***	0.730**	0.694*	0.493	0.384	0.133	0.192

*, *p* <0.05;

**, *p* <0.01;

***, *p* <0.001.

Contrary to the observations for the bacterial community diversity, no significant differences in fungal community richness and *H*′ between soil types and between soil horizons were observed (Table 1). Altitude had a significant positive influence on the fungal community richness and *H*′ in the O horizon (*p* <0.05; Table 2). Fungal *H*′ was significantly positively correlated with lignin content (*p* <0.05) and negatively correlated with water-soluble polysaccharide content (*p* <0.05) in the O horizon (Table 2). No significant influence of soil properties on the fungal community diversity was observed in the A horizon (Table 3).

**Fig 1:**
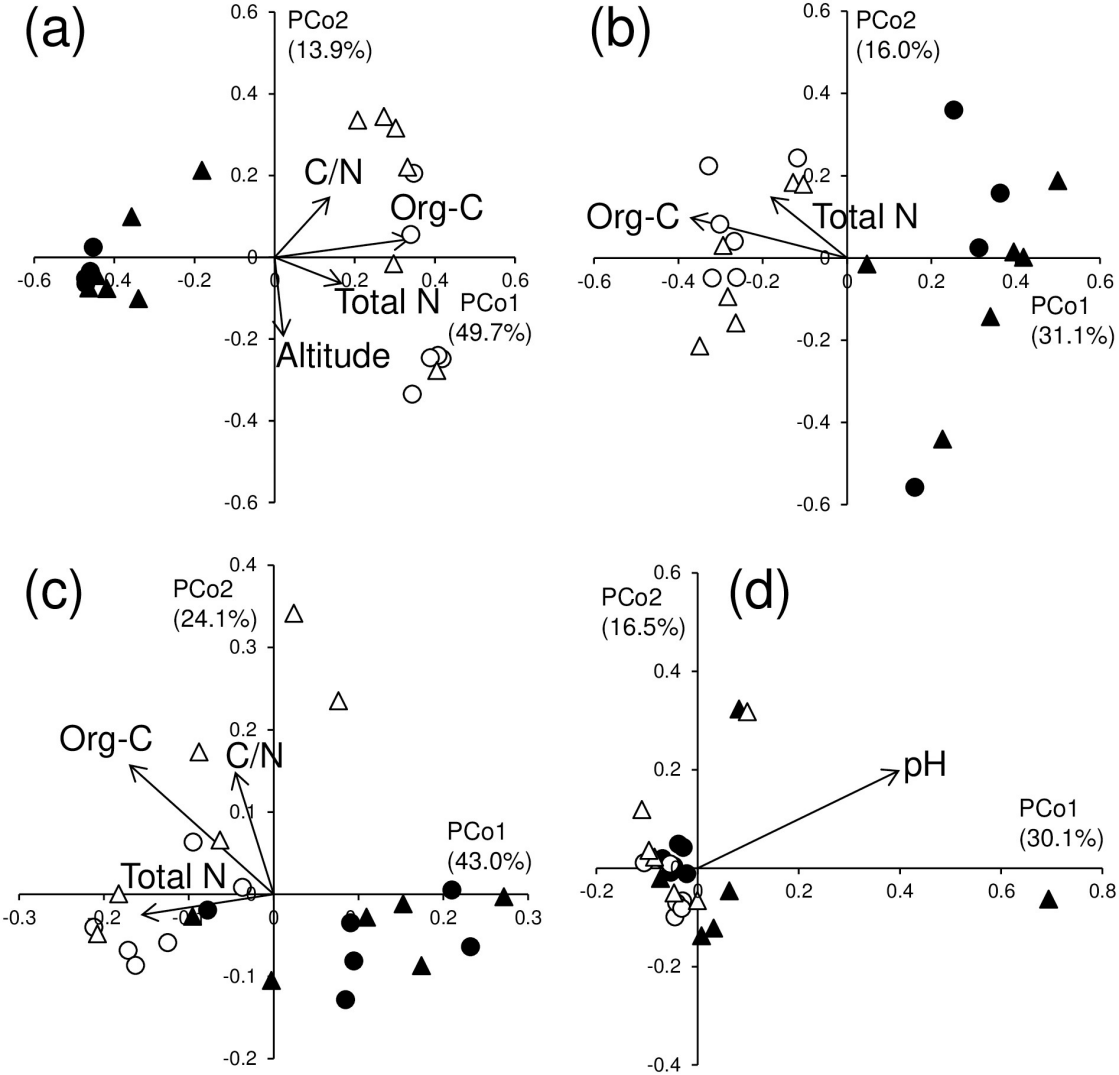
Principal coordinate analysis ordination of (a) bacterial DGGE profiles, (b) fungal DGGE profiles, (c) bacterial CLPP using the ECO plate, and (d) fungal CLPP using the SFN2 plate in the O and A horizons of Andosols and Cambisols. Filled triangle: A horizon of Cambisols; filled circle: A horizon of Andosols; hollow triangle: O horizon of Cambisols; hollow circle: O horizon of Andosols. Vectors for the significantly correlated (*p* < 0.1) environmental variables are superimposed on the plot.

**Fig 2:**
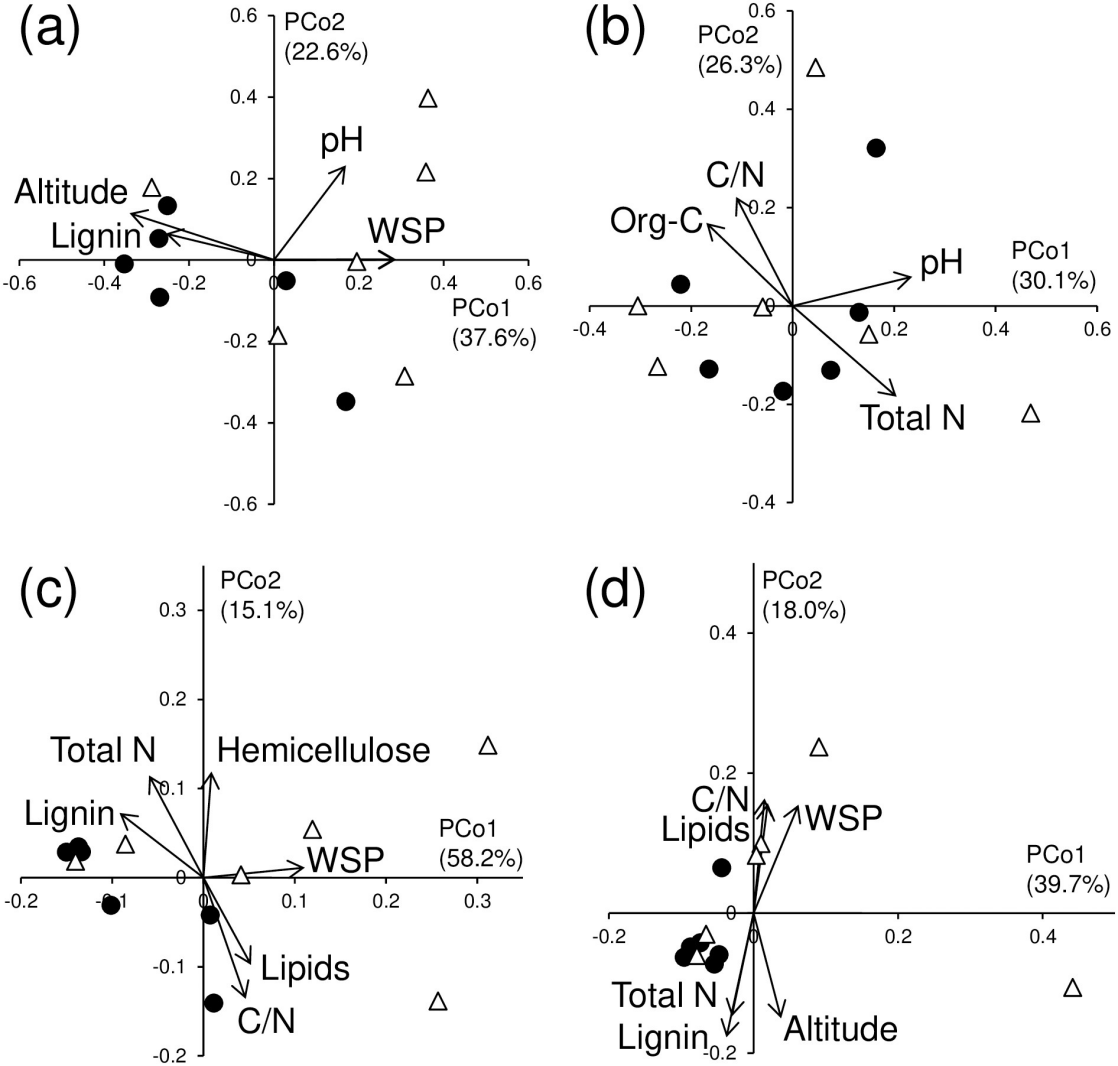
Principal coordinate analysis ordination of (a) bacterial DGGE profiles, (b) fungal DGGE profiles, (c) bacterial CLPP using the ECO plate, and (d) fungal CLPP using the SFN2 plate in the O horizon of Andosols and Cambisols. Filled circle: Andosols; hollow triangle: Cambisols. WSP: water-soluble polysaccharides. Vectors for the significantly correlated (*p* < 0.1) environmental variables are superimposed on the plot.

**Fig 3:**
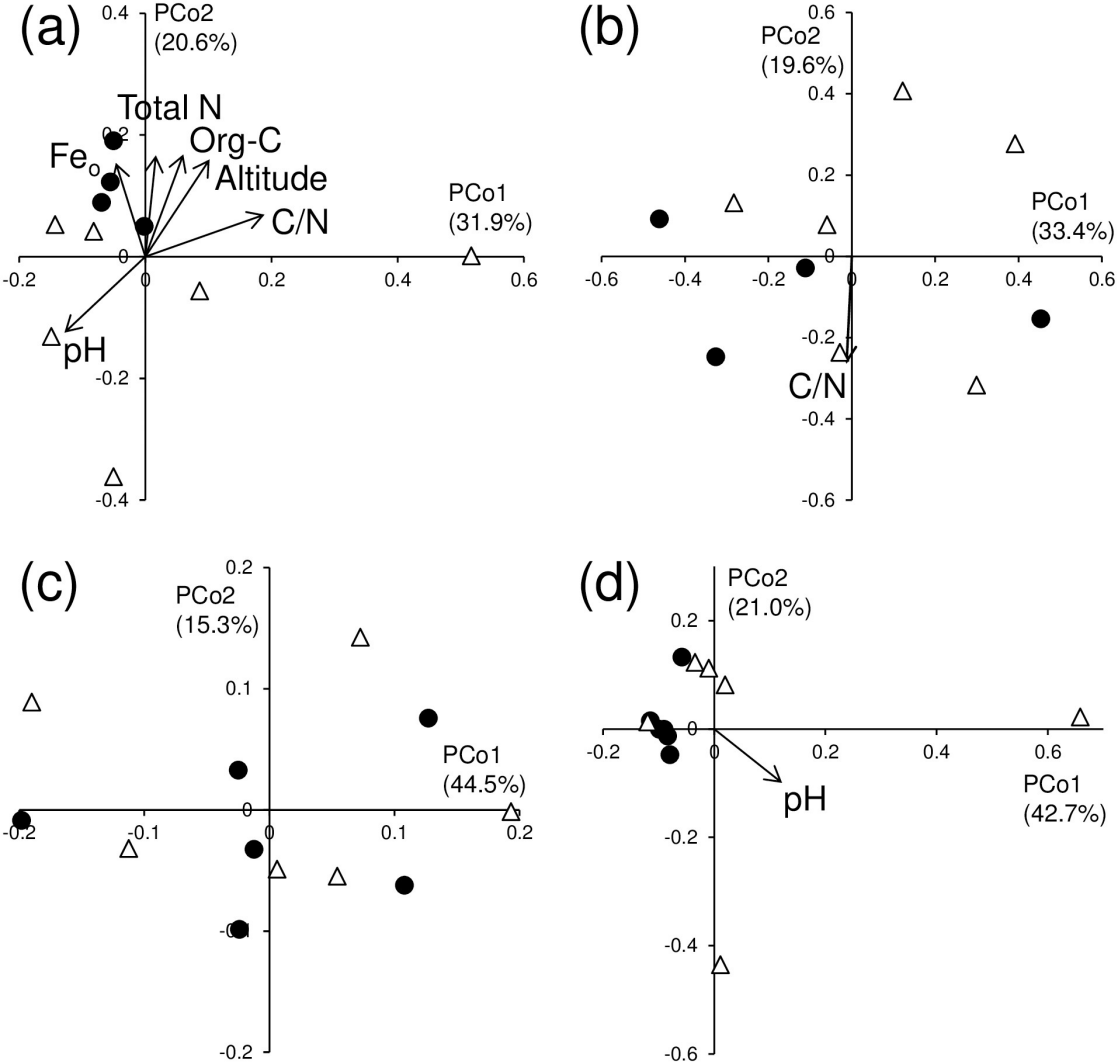
Principal coordinate analysis ordination of (a) bacterial DGGE profiles, (b) fungal DGGE profiles, (c) bacterial CLPP using the ECO plate, and (d) fungal CLPP using the SFN2 plate in the A horizon of Andosols and Cambisols. Filled circle: Andosols; hollow triangle: Cambisols. Vectors for the significantly correlated (*p* < 0.1) environmental variables are superimposed on the plot.

In the PCoA ordination, the DGGE profiles were clearly divided between the O and A horizons for both bacterial (Fig 1a) and fungal (Fig 1b) communities. In both horizons, explicit distinction between the soil types for both microbial communities was not observed (Figs 2a and 2b, 3a and 3b). Altitude and pH influenced the bacterial DGGE profiles in common with the O and A horizons (Figs 2a and 3a), whereas a significant influence of C/N ratio on the fungal DGGE profiles was discerned in both horizons (Figs 2b and 3b).

### BIOLOG analysis of bacterial and fungal communities

The AWCD and metabolic diversity, *H*′, of the bacterial community based on a BIOLOG ECO plate analysis exhibited no significant differences between the soil types and between the soil horizons (Table 1). Organic C (*p* <0.05) and water-soluble polysaccharide contents (*p* <0.05) negatively affected the bacterial AWCD, whereas lignin content (*p* <0.05) was positively correlated with the bacterial AWCD in the O horizon (Table 2). Water-soluble polysaccharide content negatively affected potential functional diversity, *H*′, of the bacterial community in the O horizon (*p* <0.05). No significant influence of litter properties on AWCD and potential functional diversity of the fungal community was observed in the O horizon (Table 2). In the A horizon, no soil properties were significantly correlated with AWCD and metabolic diversity of the bacterial and fungal communities other than a significant negative influence of soil pH on the fungal potential functional diversity (*p* <0.05; Table 3). It is noteworthy that microbial biomass C was not significantly correlated with AWCD and metabolic diversity of the bacterial and fungal communities.

In the PCoA ordination, the bacterial CLPP tended to be separated between the O and A horizons (Fig 1c), but such a trend was not observed for the fungal CLPP (Fig 1d). In each of the O and A horizons, no distinct difference was detected between the soil types for both microbial CLPPs (Figs 2c and 2d, 3c and 3d). In the O horizon, the same indicators of nutrient status for litter (i.e., C/N ratio, lignin, lipids, total N, and water-soluble polysaccharide contents) significantly affected the CLPP for the bacterial and fungal communities (Figs 2c and 2d). In the A horizon, all soil properties examined did not influence the CLPP for both bacterial and fungal communities other than pH affecting the fungal CLPP (Fig 3c and 3d).

## Discussion

Bacterial CLPP, and bacterial and fungal community composition showed distinct differences between the O and A horizons in the PCoA (Fig 1). In addition, the bacterial species richness and *H*′ based on the DGGE profile were significantly greater in the A horizon than in the O horizon (*p* <0.05; Table 1). These findings supported Hypothesis 1 that microbial community composition and potential functioning differ between the O and A horizons. This might be attributed to the fact that the composition of organic matter, the substrate of microorganisms, largely differs between plant litter in the O horizon (mainly cellulose, lignin, and hemicellulose) [36] and soil organic matter in the A horizon (primarily humic substances) [36]. According to Kirchman, high proportions of cellulose and hemicellulose would select for certain bacteria in the O horizon [37], which would lower the bacterial species richness and *H*′ in the O horizon and differentiate the microbial community composition and potential functioning between the two horizons. However, with regard to the fungal CLPP, no distinct difference was observed between the O and A horizons in the PCoA (Fig 1d). We do not have a reasonable explanation for the fungal CLPP result.

With respect to the bacterial DGGE profiles in the O and A horizons (Figs 2a and 3a), the pH, nutrient status (e.g., water-soluble polysaccharide, lignin, organic C, and total N contents, and C/N ratio), and altitude had significant influences. Significant effects of nutrient status and, to a greater extent, pH on bacterial community composition have been reported by several studies [3–5, 38–40]. For the fungal community, the C/N ratio was a common driver that shaped the community composition in both horizons (Figs 2b and 3b). Lauber et al. [3] and Bao et al. [40] also observed a significant effect of C/N ratio on soil fungal community composition.

For the bacterial and fungal CLPPs, several nutrient status indicators had significant influences in the O horizon (Fig 2c and 2d), but such effects were not observed in the A horizon (Fig 3c and 3d). Similarly, Klimek et al. reported that the bacterial CLPP was influenced by nutrient status in the O horizon, but such an effect was not observed in the A horizon in temperate forest soils [41]. In contrast, significant effects of nutrient status on the bacterial CLPP were observed in pine forest soil [5] and Mediterranean forest soils [42]. Hence, the contribution of nutrient status to the CLPP is likely to vary between sites, and factor(s) influencing the CLPP might be site-specific. No strong influence of soil type on the DGGE profiles and the CLPP was detected in either the O or A horizons (Figs 2 and 3). This result differed from previous observations for arable soils where the soil type was the primary determinant of bacterial community composition [26, 43]. This inconsistency might be ascribed to the greater variation in the litter and soil properties between sites in forests than in arable fields. It should be noted that although mycorrhizal fungi would play important roles in forests, our approach used in this study was not able to specify their composition and potential functioning.

We used Procrustes analysis to explore the link between the O and A horizons for the DGGE profiles and for the CLPPs, but observed no significant correlations between the horizons (Table 4). This observation did not substantiate Hypothesis 2 that differences in the O horizon between sites are accompanied by corresponding differences in the A horizon between sites for both community composition and potential functioning. The present results suggested that different factors had considerable influences on microbial communities between the O and A horizons. For example, for the fungal DGGE profiles, significant influences of total N, organic C, and pH were apparent only in the O horizon (Figs 2b and 3b).

**Table 4:** Results of Procrustes analysis comparing PCoA ordinations for microbial properties between O and A horizons.

	*M* ^2^	*r*	*p*
16S rDNA DGGE (*n* = 10)	0.725	0.525	0.159
18S rDNA DGGE (*n* = 10)	0.934	0.257	0.851
ECO plate for bacteria (*n* = 12)	0.734	0.516	0.091
SFN2 plate for fungi (*n* = 12)	0.841	0.398	0.228

Interestingly, Procrustes analysis revealed significant couplings between the bacterial and fungal communities for both community composition and potential functioning, i.e., bacterial DGGE profile−fungal DGGE profile (*p* <0.05 for O horizon; *p* <0.01 for A horizon) and bacterial CLPP−fungal CLPP (*p* = 0.001 for O horizon; *p* <0.01 for A horizon) in each of the O and A horizons (Table 5). In addition, the potential functional diversity (i.e., *H*′ based on the BIOLOG results) of the bacterial and fungal communities was significantly correlated in each horizon (*p* <0.001 for O horizon and *p* <0.01 for A horizon; Tables 2 and 3). Coelho et al. reported a significant congruence between bacterial and microeukaryotic PCoA ordinations based on pyrosequencing data for 16S/18S rDNA in several sediment samples [44]. Singh et al. reported a significant correlation between bacterial and fungal terminal restriction fragment length polymorphisms in a Procrustes analysis of grassland soils [45]. A possible explanation for the present result is that common factors had significant influences on the profiles of both bacterial and fungal communities: pH in the bacterial and fungal DGGE profiles (Fig 2a and 2b) and total N, C/N ratio, lignin, and lipids in the bacterial and fungal CLPP (Fig 2c and 2d) in the O horizon, and C/N ratio in the bacterial and fungal DGGE profiles (Fig 3a and 3b) in the A horizon (no common factors were detected for the bacterial and fungal CLPPs in the A horizon). It should be noted that there will be non-considered factors that explain microbial community composition and potential functioning, especially for the A horizon, in the present study because, contrary to the results for the O horizon (Fig 2), most of the examined factors had no significant influence on the DGGE and CLPP except for the bacterial DGGE profile in the A horizon (Fig 3).

**Table 5:** Results of Procrustes analysis comparing PCoA ordinations between microbial properties within each soil horizon.

		*M* ^2^	*r*	*p*
O horizon				
	16S rDNA— 18S rDNA (*n* = 12)	0.686	0.561	0.046
	16S rDNA—Bacterial CLPP (*n* = 12)	0.967	0.181	0.903
	18S rDNA—Fungal CLPP (*n* = 12)	0.765	0.485	0.158
	ECO—SFN2 (*n* = 12)	0.232	0.876	0.001
A horizon				
	16S rDNA— 18S rDNA (*n* = 10)	0.453	0.740	0.002
	16S rDNA—Bacterial CLPP (*n* = 10)	0.539	0.679	0.006
	18S rDNA—Fungal CLPP (*n* = 10)	0.800	0.448	0.349
	Bacterial CLPP—Fungal CLPP (*n* = 12)	0.587	0.643	0.005

Although a significant coupling was observed between the bacterial community composition and potential functioning (i.e., bacterial DGGE profile−bacterial CLPP) in the A horizon (*p* <0.01; Table 5), such a result was not obtained for the fungal community (i.e., fungal DGGE profile−fungal CLPP) in the A horizon and for both bacterial and fungal communities in the O horizon (Table 5). In addition, the genetic diversity (i.e., species richness and *H*′ based on the DGGE profile) were not significantly correlated to the potential functional diversity (*H*′ based on the BIOLOG results) for bacterial and fungal communities in the O and A horizons (Tables 2 and 3). These results did not support Hypothesis 3 that potential functioning of the microbial community is associated with the community composition. The present observations would be affected, probably to a substantial degree, by the fact that the BIOLOG results reflect only rapidly growing microorganisms, whereas cultivation-independent DGGE analysis reflects the entire microbial community in the soil. However, in a previous cluster analysis, bacterial DGGE and bacterial CLPP results were similar for tea plantation and forest soils [46]. Furthermore, strong couplings have been reported for bacterial CLPP and phospholipid fatty acid profiles in a montane area [47], and bacterial CLPP and bacterial community composition in pitcher plant microcosms [48]. Further research is warranted to understand the correspondence between microbial community composition and potential functioning.

## Conclusions

Both the CLPP and DGGE profile were clearly separated between the O and A horizons in PCoA ordinations except for the fungal CLPP. No significant couplings of the CLPP between the O and A horizons and of the DGGE profile between the two horizons were detected by Procrustes analysis for both bacterial and fungal communities. Also, no significant coupling was observed between the community composition and the potential functioning except for the bacterial community in the A horizon. In addition, the community diversity was not associated with the potential functional diversity for the bacterial and fungal communities. Unexpectedly, significant links between the bacterial and fungal DGGE profiles and between the bacterial and fungal CLPPs were observed in each of the O and A horizons. The present results do not fully unravel the factors that shape the composition and potential functioning of microbial communities in forest soils, especially for the A horizon, and further studies are warranted to elucidate the factors. Nevertheless, the present study clearly showed that different factors have substantial influences on microbial communities between the O and A horizons but that common factors affect both the bacterial and fungal communities in each horizon. These results have important implications for C and nutrient cycling in forest soils rich in organic matter.
